# Phosphorylation of IGFBP-3 by Casein Kinase 2 Blocks Its Interaction with Hyaluronan, Enabling HA-CD44 Signaling Leading to Increased NSCLC Cell Survival and Cisplatin Resistance

**DOI:** 10.3390/cells12030405

**Published:** 2023-01-25

**Authors:** Kai-ling Coleman, Michael Chiaramonti, Ben Haddad, Robert Ranzenberger, Heather Henning, Hind Al Khashali, Ravel Ray, Ban Darweesh, Jeffrey Guthrie, Deborah Heyl, Hedeel Guy Evans

**Affiliations:** Chemistry Department, Eastern Michigan University, Ypsilanti, MI 48197, USA

**Keywords:** Casein Kinase 2, CD44, IGFBP-3, lung cancer, cisplatin, hyaluronan, p53, signaling, phosphorylation, extracellular

## Abstract

Cisplatin is a platinum agent used in the treatment of non-small cell lung cancer (NSCLC). Much remains unknown regarding the basic operative mechanisms underlying cisplatin resistance in NSCLC. In this study, we found that phosphorylation of IGFBP-3 by CK2 (P-IGFBP-3) decreased its binding to hyaluronan (HA) but not to IGF-1 and rendered the protein less effective at reducing cell viability or increasing apoptosis than the non-phosphorylated protein with or without cisplatin in the human NSCLC cell lines, A549 and H1299. Our data suggest that blocking CD44 signaling augmented the effects of cisplatin and that IGFBP-3 was more effective at inhibiting HA-CD44 signaling than P-IGFBP-3. Blocking CK2 activity and HA-CD44 signaling increased cisplatin sensitivity and more effectively blocked the PI3K and AKT activities and the phospho/total NFκB ratio and led to increased p53 activation in A549 cells. Increased cell sensitivity to cisplatin was observed upon co-treatment with inhibitors targeted against PI3K, AKT, and NFκB while blocking p53 activity decreased A549 cell sensitivity to cisplatin. Our findings shed light on a novel mechanism employed by CK2 in phosphorylating IGFBP-3 and increasing cisplatin resistance in NSCLC. Blocking phosphorylation of IGFBP-3 by CK2 may be an effective strategy to increase NSCLC sensitivity to cisplatin.

## 1. Introduction

Worldwide, the leading cause of cancer-related deaths in both men and women is lung cancer with non-small cell lung cancer (NSCLC) accounting for ~80% of all lung cancer mortality [1].

The small-molecule platinum compound, cisplatin, among other platinating compounds is part of the platinum-based antineoplastic family of medications [2,3]. In general, it exerts its cytotoxic effects via the generation of DNA-platinum adducts, inducing a DNA damage response in tumor cells [4]. Cisplatin is a pivotal genotoxic chemotherapy drug with clinical activity against a variety of tumors, especially lung cancers [5]. It has been used for the treatment of patients with different types of cancer including lung for decades causing tumor death through proapoptotic mechanisms [3]. Cisplatin sensitivity was shown to increase in NSCLC cells by regulating epithelial-mesenchymal transition via inhibition of eukaryotic translation initiation factor 5A2 [4]. Despite positive initial responsiveness to cisplatin-based chemotherapy, however, relapse frequently occurs due to development of cisplatin resistance in a majority of cancer patients, resulting in a marked impediment in its clinical effectiveness [3]. Recently, we reported findings on how nicotinic acetylcholine receptors, tropomyosin receptor kinase B, and β-adrenergic receptors lead to regulation of EGFR and IGF-1R in NSCLC to affect phosphatidylinositol 3-kinase (PI3K)/AKT signaling and chemoresistance [6].

Casein Kinase II (CK2) is a ubiquitous, constitutively active serine/threonine protein kinase in eukaryotes that is usually found as a heterotetrameric complex consisting of two catalytic and two regulatory subunits [7]. The number of proteins, including extracellular matrix molecules, found to be phosphorylated is increasingly realized [8,9]. CK2 plays a multifunctional role and is considered to be a major contributor to the generation of the human phospho-proteome [7]. It participates in phosphorylation of hundreds of proteins and the regulation of a variety of signaling cascades promoting growth, invasion and metastasis in a range of human cancers, including lung cancer [7]. Moreover, the enzyme is well-known to function as a key potent suppressor of apoptosis, and blocking its activity led to apoptosis induction in cancer cells [10]. CK2 holoenzyme was found to be exported to the external side of the cellular membrane [11]. and shown to accumulate in functional form in the conditioned cell culture media of different cell lines and in cancer sera [11,12,13,14]. While the expression levels of CK2 are well-regulated in normal cells, overexpression of CK2 is frequently found in a variety of human cancers, including lung, and can lead to the development of tumors [15]. Inhibition of CK2 in combination with drugs such as cisplatin was shown to have a synergistic effect in decreasing tumor growth in preclinical models of cancer [16].

The glycosaminoglycan, hyaluronan (HA), is an anionic, non-sulfated glycosaminoglycan [17,18,19,20]. polymer that consists of the disaccharide sequence (D-glucuronic acid and D-N-acetylglucosamine) [20,21,22]. HA is an important component of the extracellular matrix (ECM), that along with its major cell surface receptor, CD44, promotes tumor progression in a variety of malignant neoplasms [18,19,21,23,24,25,26]. The steady state levels of HA are known to be minimal in most normal tissues while HA production and its accumulation in the tumor parenchyma have been reported to occur in certain types of cancer, such as lung cancer, and correlate with enhanced proliferation and metastasis and poor clinical outcomes [18,21,22,23,26]. HA has been linked to treatment resistance to several chemotherapeutic drugs, such as cisplatin [18,19,21,23,27]. HA was suggested to play a role in creating a permissive environment for tumor growth and metastasis [28]. HA has been reported to act as a biological barrier blocking delivery of anticancer agents to cancer cells by preventing their diffusion and penetration into the tumor tissue [28]. Treatment with 4-Methylumbelliferone (4-MU), which inhibits HA synthases, blocked HA accumulation in the ECM, decreased CD44 activation, resulted in diminished PI3K, AKT, and ERK signaling, and led to proapoptotic, antiproliferative, and antimetastatic effects in cultured tumor cells [28,29,30,31]. These effects could be rescued by addition of HA to cultured cells, suggesting that these effects are a direct result of a decrease in HA synthase activity [28,30,32]. A combined treatment of 4-MU and 5-fluorouracil was found to increase the effectiveness of 5-fluorouracil on pancreatic cancer cell proliferation than treatment with 5-fluorouracil alone [32]. 

Insulin-like growth factor binding protein 3 (IGFBP-3) belongs to a family that consists of six insulin-like growth factor 1 (IGF-1) binding proteins comprised of three distinct (N-terminal, linker, and C-terminal) domains [33,34,35,36,37,38]. IGFBP-3 is the most abundant IGFBP and the main carrier of IGF-1 in the circulation [33,36,38]. It can bind IGF-1 with high affinity, attenuating IGF/IGF-1R interactions, leading to antiproliferative effects [33,36,38]. IGFBP-3 is also known to operate independently of the IGF/IGF-1R axis to regulate cell survival [38,39,40,41]. Lower expression of IGFBP-3 [42]. in lung cancer is associated with poor diagnosis in patients with stage I NSCLC [43,44,45,46,47]. An inverse relationship between plasma or serum levels of IGFBP-3 and lung cancer risk, has been previously reported [33,38,48]. Diminished survival of human lung cancer cells was correlated with increased expression of IGFBP-3 [49]. Our previous studies showed that IGFBP-3 binds HA via the 18-residue basic domain, amino acid residues 215–232 (^215^-KKGFYKKKQCRPSKGRKR-^232^) in the C-terminal region of the protein, abolishing HA-CD44 interactions, and decreasing A549 lung cancer cell viability [50]. In addition, we showed that IGFBP-3 likely exerts its cytotoxic effects on cell survival via a mechanism that depends on HA-CD44 interactions since co-treatment with the anti-CD44 antibody (5F12), known to block HA-CD44 binding, and IGFBP-3 did not result in an additive negative effect on cell viability [50]. Lack of IGFBP-3 expression due to promoter hypermethylation decreased sensitivity of NSCLC cells to cisplatin [51]. 

In this study, we investigated a molecular mechanism underlying cisplatin resistance in two NSCLC cell lines, A549 (p53 wild-type) and H1299 (p53-null) and show that CK2, CD44, and IGFBP-3 are important regulators of cisplatin-induced apoptosis in human lung cancer cells.

CK2 is well-documented as an anti-apoptotic kinase that sustains cell survival [14,16,52,53]. The mechanisms underlying its functions, however, are several and only partially known [7,52,54]. In this study, we identify a novel mechanism employed by CK2 in regulating cell survival in NSCLC cells. Our results show that phosphorylation of IGFBP-3 by CK2 blocks the binding of IGFBP-3 to HA, activating HA-CD44 signaling, resulting in decreased apoptosis, increased cell survival, and cisplatin resistance. The findings of this study add a new mechanism to the multitude of diverse mechanisms known to be employed by CK2 in potentiating cell survival signaling.

## 2. Materials and Methods

### 2.1. Materials

Most of the material used in this study was purchased as we reported earlier [50,55,56,57,58,59]. Phosphate Buffered Saline (PBS), nitrocellulose membranes, streptavidin-horseradish peroxidase (HRP) conjugate, Ponceau S solution, LY294002 hydrochloride, hydrogen peroxide solution, AKT Inhibitor (Calbiochem, San Diego, CA, USA), pifithrin-α p-Nitro, ATP, cisplatin, biotin-HA (B1557), and Anti-IGF-1 antibody produced in goat (SAB2501424) were purchased from Sigma-Aldrich. The selective cell-permeable CK2 inhibitor (4,5,6,7-tetrabromobenzotriazole, TBB) was from R&D Systems. Nonglycosylated recombinant IGFBP-3 (MBS142177) was from MyBioSource (San Diego, CA, USA). Casein Kinase II (CK2, P6010S) was purchased from New England Biolabs (Ipswich, MA, USA). The caspase 3 (cleaved) colorimetric In-Cell ELISA Kit (62218), CD44 antibody (5F12, MA5-12394), IGFBP3 Antibody (PA5-18791), mouse α-tubulin monoclonal antibody (DM1A), active caspase 3 recombinant rabbit monoclonal antibody (SR01-02), goat anti-rabbit IgG (H+L) secondary antibody (HRP, 31466), mouse IgG isotype control, (mIgG), 3,3′,5,5′-tetramethylbenzidine (TMB), BCA protein assay kit, super signal west pico luminol (chemiluminescence) reagent, and the Halt Protease and Phosphatase Inhibitor Cocktail were from ThermoFisher (Waltham, MA, USA). The broad-spectrum protein kinase inhibitor, staurosporine, was purchased from Tocris (Minneapolis, MN, USA). Recombinant human IGF-1 protein (ab270062), donkey anti-mouse IgG (HRP) (ab205724), and rabbit anti-goat IgG H&L (HRP) (ab6741) were purchased from Abcam (Waltham, MA, USA). Anti-phosphoserine antibody (16B4, sc-81514), m-IgGκ BP-HRP, and nuclear factor kappa B (NFκB) inhibitor (CAS 213546-53-3) were from Santa Cruz Biotechnology (Dallas, TX, USA). 

### 2.2. Cell Culture

The human NSCLC cell lines, A549 (ATCC CCL-185) and H1299 (ATCC CRL-5803), were purchased from the American Type Culture Collection (ATCC, Manassas, VA, USA). Cells were seeded as we reported earlier [50,55,56,57,58,60,61]. in 5 mL HyClone Dulbecco’s modified Eagle’s media/nutrient mixture F-12 (DMEM/F12) (GE Healthcare Life Sciences, Pittsburgh, PA, USA), supplemented with 10% Fetalgro bovine growth serum (FBS, RMBIO, Missoula, MT, USA), 50 U/mL penicillin, and 50 U/mL streptomycin (Invitrogen Life Technologies, Carlsbad, CA, USA) in 25 cm^2^ tissue culture flasks, and allowed to grow overnight in an incubator at 37 °C, 95% humidity, and 5% CO_2_. The cells were counted after trypan blue staining, with a hemocytometer. 

When inhibitors were used, cells were treated with inhibitors targeted against CK2 (TBB, 7.5 µM), PI3K (LY294002, 14.5 μM), AKT (AKT inhibitor, 1.75 μM), NFκB (NFκB inhibitor, 18 μM), and p53 (pifithrin-α, 10 μM). 

### 2.3. MTT Assay

The MTT reduction assay (Sigma-Aldrich, Burlington, MA, USA), used to measure cell viability, was carried out as we reported earlier [50,57,62]. The absorbance was measured at 570 nm in a plate reader. All absorbance measurements were in the linear range. Untreated cells or wells containing only DMSO and media were used as a positive and negative control, respectively. Cell treatment with purified IGFBP-3 protein, non-phosphorylated or phosphorylated in vitro with CK2, was carried out in the presence of a mixture of EDTA-free phosphatase and protease inhibitors as was previously carried out [13,63,64]. and a broad-spectrum kinase inhibitor. 

### 2.4. Apoptosis Assay

For the caspase 3 (cleaved) colorimetric assay, activated (cleaved) caspase 3 and tubulin, were simultaneously measured in triplicate in whole cells by an in-cell ELISA assay (62218, ThermoFisher) using 96-well microplates as we previously described [62,65]. Control cells were treated with a 0.1% DMSO vehicle control and contained all the reagents except the primary antibodies. The average of all replicate nonspecific background signal controls from each condition was subtracted then the average absorbance at 450 nm for each condition was calculated. Cell treatment with purified IGFBP-3 protein, non-phosphorylated or phosphorylated in vitro with CK2, was carried out in the presence of a mixture of EDTA-free phosphatase and protease inhibitors as was previously carried out [13,63,64]. and a broad-spectrum kinase inhibitor. 

### 2.5. ELISA

ELISAs were conducted as we reported previously using Nunc MaxiSorp 96-well Flat Bottom plate (ThermoFisher) wells [50,57,58,66]. All absorbance measurements were in the linear range. To monitor non-specific binding, negative control wells on the plates included, for example, bound pure IGFBP-3 or P-IGFBP-3, then adding all components, streptavidin-horseradish peroxidase and TMB, but without addition of biotin-HA. Before analysis, the OD from the data was corrected for non-specific binding by subtracting the mean background absorbance for the negative controls. Statistical analysis was determined by the GraphPad Prism 9.4.1 software. (San Diego, CA, USA) Data were expressed as the mean ± S.D. Three to five independent experiments were carried out in triplicate for each assay condition. 

### 2.6. Western Blotting

Samples of the cell lysates were analyzed according to our previous protocols [58,59,67]. Briefly, attached live cells were harvested and the cell pellet was resuspended in 1 mL lysis buffer consisting of 20 mM Tris/HCl, pH 7.5, 137 mM NaCl, 1% triton X-100, 10% glycerol, 1 mM PMSF, and Halt protease and phosphatase inhibitor cocktail (ThermoFisher). Samples were briefly sonicated, centrifuged and the supernatants were stored at −80 °C. The BCA protein assay kit was used to measure the protein concentration. Following the methods that we reported previously [59,67]., samples were fractionated by SDS-PAGE on a 12% gel then transferred to a nitrocellulose membrane. The membrane was blocked in TBST buffer, pH 7.6, containing 5% nonfat milk for 6 h at 4 °C, incubated with the primary and secondary antibodies, developed using SuperSignal West Pico luminol (chemiluminescence) reagent, and imaged with a Bio-Rad molecular imager. Bands were quantitated using Image J 1.47 v software (U. S. NIH, Bethesda, MD, USA).

### 2.7. PI3K Assay

Activated phosphorylated-PI3K p85+ total PI3K p85 in-cell ELISA kit (Abcam) was used according to the recommendations by the manufacturer as we recently reported [58,61]. Briefly, cells were cultured in 96-well plates then treated as indicated. Following treatment, the cells were fixed, and the wells were then incubated with a primary antibody targeting either total PI3K p85 (recognizes the total level of PI3K p85 proteins regardless of the phosphorylation state) or phosphorylated-PI3K p85 (recognizes p85 PI3K alpha/gamma phospho-tyrosine 467/199). Secondary HRP-conjugated antibodies were then added, and the signal detected after addition of the developing solution. Crystal Violet solution was then added to determine the relative number of cells in each well. Signals for phospho-PI3K and total-PI3K were normalized to cell number then the ratio of phospho-PI3K to total-PI3K for each treatment was determined and plotted. 

### 2.8. AKT assay

The AKT kinase activity assay kit (Abcam) was used to quantitate the activity of AKT according to the manufacturer’s instructions as we reported earlier [58,61]. In brief, the assay is based on a solid phase ELISA. A specific synthetic peptide is used as a substrate for AKT along with a polyclonal antibody that binds the phosphorylated substrate. 

### 2.9. NFκB Assay

The NFκB p65 (Phospho/Total) InstantOne sandwich ELISA kit (Invitrogen) was used as we reported previously [58,61]. according to the manufacturer’s recommendations. Signals for phospho (Ser536) and total NFκB were normalized to cell number, then the ratio of phospho (Ser536) to total NFκB for each treatment was determined and plotted. 

### 2.10. p53 Transcription Factor Activity Assay

The activity of p53 was assayed using the colorimetric BioVision’s p53 transcription factor activity assay (Catalog # K923-100) kit. Briefly, a 96-well plate is coated with double stranded oligonucleotides. Cell lysates containing activated p53 were then added to the wells allowing interaction with the oligonucleotides in the plate wells. A p53 primary antibody was then added followed by addition of a HRP-conjugated secondary antibody. The color signal was developed after addition of the TMB substrate and measured at 450 nm. Another kit (RayBio, (Peachtree Corners, GA, USA) human p53 transcription factor activity assay kit (Catalog #: TFEH-p53) was also used. Briefly, 96-well plates are coated with double-stranded oligonucleotides containing the p53 binding sequence that specifically capture the active p53 contained in whole cell lysates after a brief incubation. A primary antibody against p53 recognizes the p53-DNA complex in each well, then a HRP-conjugated secondary antibody is used for detection of the signal at 450 nm. 

### 2.11. Statistical Analysis

The analysis was carried out as we previously reported [55,56,57,61]. Each experiment in this study was performed in triplicate and repeated a minimum of three times. Statistical values are expressed as the mean ± Standard Deviation (SD). To evaluate the statistical differences, the Mann–Whitney or an ordinary one-way ANOVA followed by Tukey’s post hoc multiple comparison test was performed. All the statistical tests were two-sided and a *p* value of <0.05 was considered statistically significant in all cases. GraphPad Prism (GraphPad Software, 9.4.1) was used for the statistical analysis.

## 3. Results

### 3.1. Treatment with the CK2 Inhibitor (TBB) and Cisplatin Diminished Cell Viability More Effectively than Treatment with Either TBB or Cisplatin Alone 

**Figure 1:**
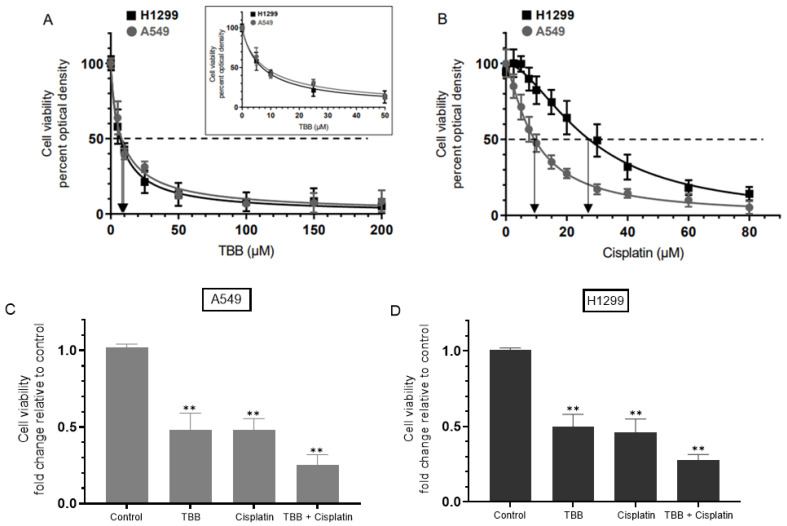
Treatment with TBB and cisplatin was more effective at decreasing cell viability compared to treatment with TBB or cisplatin alone. Cells (0.2 × 10^5^) were grown in 10% FBS-supplemented media for 24 h then serum starved overnight. The cell monolayers were then incubated in serum-free media for 72 h in the absence or presence of increasing concentrations of TBB (**A**) or cisplatin (**B**) then the cell viability was measured as described in the Methods section. Optical densities (570 nm) were normalized for the curves by expressing each point relative to the best fitted Emax value (set to 100%). The data were then plotted as a function of increasing TBB or cisplatin concentrations and fit using a nonlinear regression curve fitting approach. Viability was also measured upon treatment with TBB (7.5 µM) without or with 10 µM cisplatin when using A549 cells (**C**) and 30 µM cisplatin when using H1299 cells (**D**). Asterisks indicate a statistically significant difference from the corresponding control of each cell line, Mann–Whitney test, ** *p* < 0.01. Data were processed using the GraphPad Prism 9.4.1 software and presented as the mean ± S.D. of three independent assays, each carried out in triplicate.

Inhibition of CK2 was reported earlier to reverse cisplatin resistance in cisplatin-resistant A549 cells [53]. CK2α was shown to be a positive regulator of Notch1 signaling in A549 and H1299 lung cancer cells [68]. To examine the effect of TBB and/or cisplatin on cell viability, cells were grown in 10% FBS-supplemented media for 24 h then serum starved overnight. The cell monolayers were then incubated in serum-free media for 72 h in the absence or presence of increasing concentrations of TBB or cisplatin and then the cell viability was measured as described in the Methods section. Cell treatment with TBB (Figure 1A) decreased viability in a dose-dependent manner, both in A549 and H1299 cell lines (IC_50_ values were 8 ± 1.5 μM in A549 cells and 7 ± 1.3 μM in H1299 cells, respectively). Cell viability was also decreased by treatment with cisplatin (Figure 1B) in a dose-dependent manner with IC_50_ values for cisplatin of 9 ± 1.5 μM for A549 cells and 26 ± 4 μM for H1299 cells. These values are comparable to those we recently reported upon examining regulation of cisplatin resistance in NSCLC cells by nicotine, brain-derived neurotrophic factor (BDNF), and the β-adrenergic receptor blocker, propranolol [6]. The combined treatment of TBB and cisplatin was more potent in inhibiting cell viability than treatment with either TBB or cisplatin (Figure 1C,D). Co-incubation of A549 and H1299 cells with TBB and cisplatin resulted in ~4.00-fold decrease in cell viability as compared to ~2.00-fold decrease in viability with either TBB or cisplatin treatment (Figure 1C,D).

### 3.2. Phosphorylation of IGFBP-3 by CK2 Decreases Its Binding to HA but Not to IGF-1 

IGFBP-3 has been previously reported to be phosphorylated by CK2 [12,13]. IGFBP-3 S167 and S175 were identified as the potential sites of phosphorylation by CK2 with S167A-IGFBP-3 mutant having enhanced apoptotic functions in prostate cancer cells [12]. CK2 phosphorylation of IGFBP-3 was found to be non-essential for the ability of IGFBP-3 to bind IGF but blocked interactions of IGFBP-3 with the acid-labile subunit (ALS), [13,69]. known to bind residues 228–232 of IGFBP-3 which comprise part of the 18-residue basic domain (^215^-KKGFYKKKQCRPSKGRKR-^232^) that interacts with either humanin [70]. or HA [50,71]. Interactions of P-IGFBP-3 with the cell surface was inhibited by ~50% compared with that of the non-phosphorylated protein [13]. These studies suggested that phosphorylation might modulate interactions of IGFBP-3 with ALS and the cell surface, both of which are known to be mediated by the basic carboxyl-terminal residues of IGFBP-3 [12,13]. 

**Figure 2:**
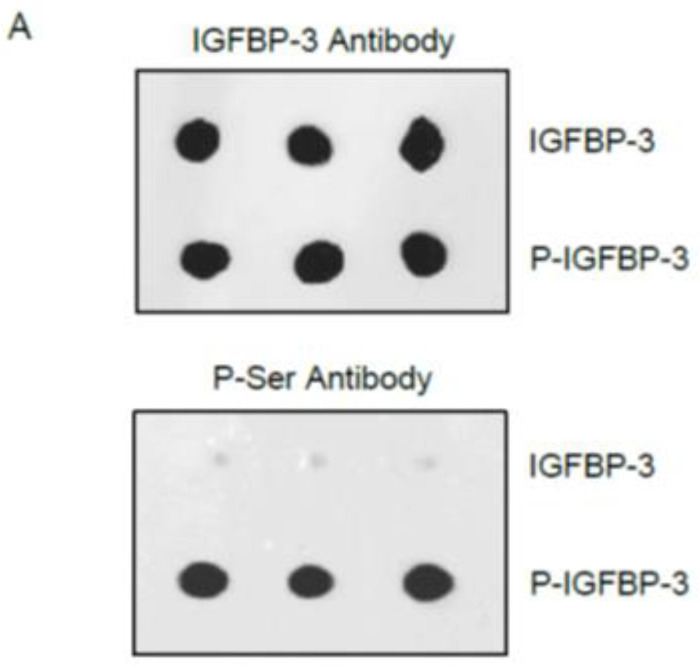

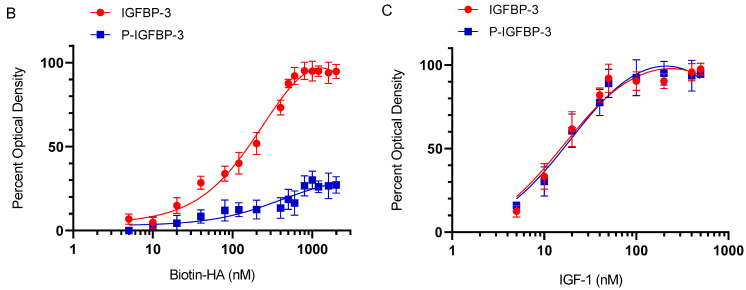
Phosphorylation of IGFBP-3 by CK2 affects its binding to HA but not to IGF-1. IGFBP-3 (50 nM) unphosphorylated or phosphorylated by CK2 (**A**) was bound to the wells, then increasing concentrations of biotin-HA (**B**) or IGF-1 (**C**) were added and processed as described in the Methods section. Optical densities (ODs, 450 nm) were normalized for the curves by expressing each point relative to the best fitted Emax value for IGFBP-3 (set to 100%). The data were then plotted as a function of increasing biotin-HA or IGF-1 concentrations and, using the GraphPad Prism 9.4.1 software, fit with a nonlinear regression curve fitting approach. Before analysis, the ODs from the data were corrected for non-specific binding by subtracting the mean background absorbance for the negative controls containing the same IGFBP-3 concentration but using water instead of biotin-HA or IGF-1. Data were expressed as the mean ± S.D. of three independent experiments, each carried out in triplicate.

To examine the ability of IGFBP-3 phosphorylated by CK2 (P-IGFBP-3) (Figure 2A) to bind either HA or IGF-1, IGFBP-3 (50 nM) was bound to the wells, then increasing concentrations of biotin-HA (Figure 2B) or IGF-1 (Figure 2C) were added and processed as described in the Methods section. Maximal binding of P-IGFBP-3 to HA was ~30% of that observed for the binding of IGFBP-3 to HA (Figure 2B) while the curves were unaltered for binding of either IGFBP-3 or P-IGFBP-3 to IGF-1 (Figure 2C). 

### 3.3. IGFBP-3 Phosphorylated by CK2 Was Less Effective than IGFBP-3 at Decreasing Cell Viability and Increasing Apoptosis in the Absence or Presence of Cisplatin

Our results (Figure 2B) showed that IGFBP-3 binding to HA was diminished upon phosphorylation of IGFBP-3 by CK2. In addition to their IGF-dependent functions, it is well-recognized that almost all the IGFBPs have IGF-independent roles [72,73]. that are less well-defined and are, in part, linked to their associations with ECM components. Earlier, we published that IGFBP-3 binds HA through residues 215–232 in the C-terminal region of the protein and blocks HA interactions with its receptor, CD44, reducing viability of A549 human lung cancer cells [50]. We then showed that IGFBP-3 or its peptide analog (215–232) blocks HA-CD44 signaling via a mechanism that depends on both p53 and acetylcholinesterase in an IGF-independent fashion [57]., and that binding of IGFBP-3 to HA was unaffected by glycosylation or reduction of IGFBP-3 [55]. We also reported that IGFBP-3 likely exerts its cytotoxic effects on cell viability through a mechanism that relies on HA-CD44 molecular interactions since using an anti-CD44 antibody (5F12) that antagonizes HA-CD44 binding in the presence of IGFBP-3 did not result in an additive negative effect on cell viability [50]. We recently found that p53 knockdown resulted in higher heparanase levels and lower IGFBP-3 levels in A549 cell media while knockdown of IGFBP-3 in A549 cells diminished p53 activity and increased the levels of heparanase in the media [67].

Preventing activation of CK2 was previously shown to enhance IGFBP-3 induced apoptosis [12], while CK2-mediated phosphorylation partially blocked apoptosis induced by IGFBP-3. To examine the role of IGFBP-3 phosphorylated by CK2 in regulating HA-CD44 signaling, cells were grown in 10% FBS-supplemented media for 24 h. The following day, the cell monolayers were incubated in serum-free media for 24 h, then treated for 72 h with cisplatin, the CD44 antibody (5F12), IGFBP-3, P-IGFBP-3, or in combination. Viability and apoptosis of A549 and H1299 cells were then assessed as described in the Methods section. 

**Figure 3:**
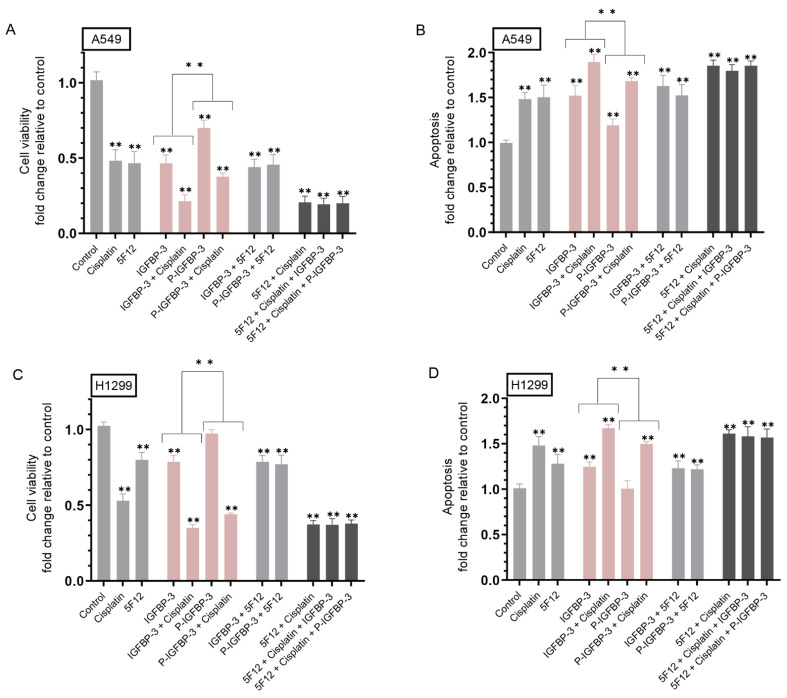
P-IGFBP-3 was less effective than IGFBP-3 at reducing cell viability and increasing apoptosis with or without cisplatin. Cells (0.2 × 10^5^) were grown in 10% FBS-supplemented media for 24 h. The following day, the cell monolayers were incubated in serum-free media overnight, then treated for 72 h with cisplatin (10 μM when using A549 cells and 30 μM when using H1299 cells), the CD44 antibody 5F12 (5 μg/mL), added either separately or 2 h prior to addition of either IGFBP-3 and/or cisplatin, IGFBP-3 or P-IGFBP-3 (50 nM), or in combination with the media containing the specific components in the various treatments replaced every 24 h. Viability and apoptosis of A549 (**A**,**B**) and H1299 (**C**,**D**) cells were then assessed as described in the Methods section. Data from five independent assays, each carried out in triplicate, were averaged, normalized, and expressed as fold change relative to untreated cells (control) using the GraphPad 9.4.1 software. The graphs summarize the results expressed as means ± SD (*n* = 5). Asterisks indicate a statistically significant difference from the corresponding control of each cell line while absence of asterisks indicates no significance, Mann–Whitney test. Statistical differences between different groups were analyzed by an ordinary one-way analysis of variance (ANOVA) followed by Tukey’s post hoc multiple comparison test. ** *p* < 0.0l.

Incubation of A549 cells with IGFBP-3 decreased viability ~2.00-fold and increased apoptosis ~1.50-fold (Figure 3A,B). Treatment of A549 cells with IGFBP-3 and cisplatin reduced viability ~4.45-fold as compared to 2.00-fold decrease measured with either IGFBP-3 or cisplatin (Figure 3A) and increased apoptosis ~1.85-fold as compared to ~1.50-fold with either treatment alone (Figure 3B). Phosphorylation of IGFBP-3 by CK2 decreased its effect on A549 cell viability and apoptosis (Figure 3A,B). Incubation with P-IGFBP-3 decreased cell viability ~1.40-fold and increased apoptosis ~1.20-fold while co-treatment with P-IGFBP-3 and cisplatin further decreased A549 cell viability by ~2.85- fold and increased apoptosis by ~1.70-fold (Figure 3A,B). Comparable trends were observed for H1299 cells (Figure 3C,D). H1299 cell treatment with IGFBP-3 decreased viability and increased apoptosis by ~1.25-fold while treatment with P-IGFBP-3 did not have a significant effect on either cell viability or apoptosis compared to control untreated cells (Figure 3C,D). Co-treatment of H1299 cells with IGFBP-3 and cisplatin diminished cell viability by ~2.75-fold as compared to ~1.25-fold decrease when using IGFBP-3 or ~2.00-fold decrease when using cisplatin (Figure 3C). These results correlated with ~1.65-fold increase in apoptosis upon co-treatment of H1299 cells with IGFBP-3 and cisplatin, an increase higher than that observed upon cell incubation with only IGFBP-3 (~1.25-fold increase) or cisplatin (~1.45-fold increase). Co-incubation of H1299 cells with P-IGFBP-3 and cisplatin decreased cell viability ~2.30-fold and increased apoptosis ~1.50-fold (Figure 3C,D). Incubation with 5F12 and cisplatin resulted in a larger decrease in A549 cell viability, ~4.75-fold, compared to ~2.00-fold decrease with either treatment alone (Figure 3A) and a greater increase in apoptosis, ~1.85-fold increase, as compared to ~1.50-fold increase with either 5F12 or cisplatin (Figure 3B). H1299 cells treated with 5F12 and cisplatin led to a ~2.70-fold decrease in cell viability as compared to treatment with either cisplatin (~2.00-fold decrease) or 5F12 (~1.25-fold decrease). These results correlated with ~1.60-fold increase in apoptosis in H1299 cells treated with 5F12 and cisplatin, ~1.25-fold increase when using 5F12, and ~1.45-fold increase when using cisplatin (Figure 3C,D). Compared to cell treatment with 5F12 and cisplatin, addition of either IGFBP-3 or P-IGFBP-3 had no effect on either cell viability or apoptosis in both cell lines (Figure 3). Collectively, these results suggest that phosphorylation of IGFBP-3 by CK2 weakens its effect on cell viability and apoptosis and its ability to augment the effects of cisplatin. 

### 3.4. Treatment of Cells with IGF-1 and Either IGFBP-3 or P-IGFBP-3 Had the Same Effect on Cell Viability and Apoptosis in the Presence of the CD44 Antibody, 5F12, with or without Cisplatin

**Figure 4:**
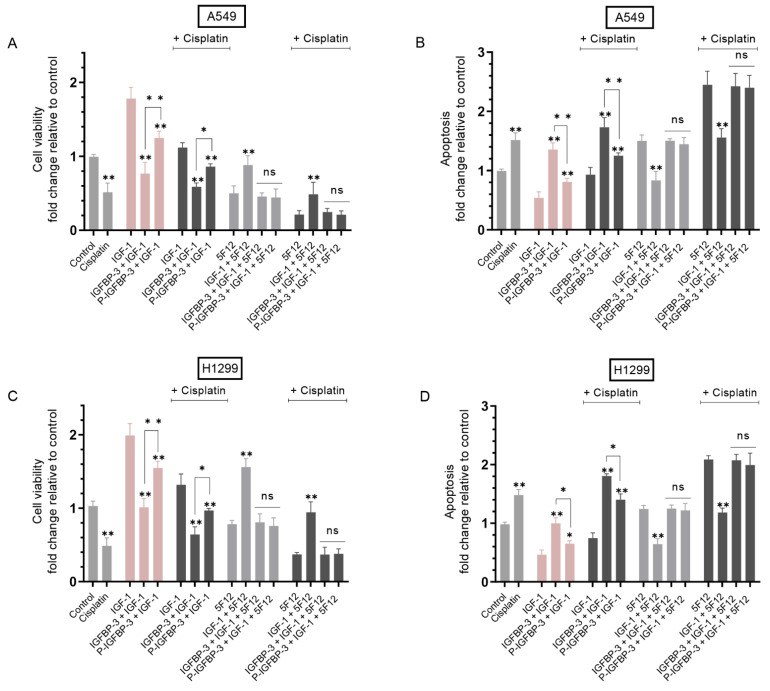
Incubation with the CD44 antibody, 5F12, abolished the differences in cell viability or apoptosis induced by cells treated with IGF-1 and either IGFBP-3 or P-IGFBP-3 with or without cisplatin. Cells (0.2 × 10^5^) were grown in 10% FBS-supplemented media for 24 h. The following day, the cell monolayers were incubated in serum-free media overnight, then treated for 72 h with cisplatin (10 μM when using A549 cells and 30 μM when using H1299 cells), IGF-1 (100 ng/mL), IGFBP-3 or P-IGFBP-3 (50 nM), the CD44 antibody 5F12 (5 μg/mL), added either separately or 2 h prior to addition of other treatments as indicated, or in combination. The media containing the specific components in the various treatments was replaced every 24 h. Viability and apoptosis of A549 (**A**,**B**) and H1299 (**C**,**D**) cells were then assessed as described in the Methods section. Data from five independent assays, each carried out in triplicate, were averaged, normalized, and expressed as fold change relative to untreated cells (control). The graphs summarize the results expressed as means ± SD (*n* = 5). Asterisks indicate a statistically significant difference from the control or single treatments of each cell line group while absence of asterisks indicates no significance using the GraphPad 9.4.1 software, Mann–Whitney test. Statistical differences between different groups were analyzed by an ordinary one-way analysis of variance (ANOVA) followed by Tukey’s post hoc multiple comparison test, * *p* < 0.05, ** *p* < 0.0l. Absence of asterisks indicates no significance (ns).

We next attempted to examine the effects of IGF-1 on cell viability in the absence or presence of IGFBP-3, P-IGFBP-3, 5F12, and cisplatin. Cells were grown in 10% FBS-supplemented media overnight. The following day, the cell monolayers were incubated in serum-free media for 24 h, then treated for 72 h with cisplatin, IGF-1, IGFBP-3, or P-IGFBP-3, the CD44 antibody 5F12, or in combination (Figure 4). Viability and apoptosis of A549 and H1299 cells were then assessed as described in the Methods section.

Treatment of A549 cells with IGF-1 resulted in ~1.75-fold increase in viability and a ~1.85-fold decrease in apoptosis relative to control untreated cells (Figure 4A,B). Similar effects were observed for H1299 cells treated under the same conditions. Treatment with IGF-1 led to ~2.0-fold increase in H1299 cell viability (Figure 4C) and ~2.15-fold decrease in apoptosis (Figure 4D).

Compared to A549 cells treated with only IGF-1, co-treatment with IGFBP-3 decreased viability ~2.30-fold and increased apoptosis ~2.50-fold. Addition of P-IGFBP-3 was less efficient at decreasing the effects of IGF-1 than IGFBP-3 since A549 cell viability decreased ~1.40-fold and apoptosis increased ~1.45-fold relative to IGF-1 treated cells. Similarly, relative to H1299 cells treated with only IGF-1, cell viability decreased upon co-treatment with IGF-1 and IGFBP-3 ~2.0-fold and ~1.25-fold with P-IGFBP-3 and IGF-1 co-treatment (Figure 4C). These results correlated with ~2.15-fold and ~1.35-fold increase in apoptosis upon H1299 cell co-treatment with IGF-1 and IGFBP-3 or IGF-1 and P-1GFBP-3, respectively, compared to H1299 cells treated with only IGF-1 (Figure 4D). While treatment of A549 and H1299 cells with IGF-1 and IGFBP-3 or P-IGFBP-3 in the presence of cisplatin further reduced cell viability and increased apoptosis, the trends were comparable to those in the absence of cisplatin (Figure 4). 

Our results (Figure 2C) show that phosphorylation of IGFBP-3 by CK2 does not alter its ability to bind IGF-1, therefore, the observed effect on cell viability or apoptosis must involve other mechanisms at play besides binding of IGF-1 to IGFBP-3/P-IGFBP-3. To unravel potential mechanisms, cells were next treated with the antibody 5F12 to examine the effect of blocking CD44 signaling on viability or apoptosis of A549 and H1299 cells treated with IGF-1, and either IGFBP-3 or P-IGFBP-3. Co-treatment of A549 cells with 5F12 and IGF-1 led to 1.75-fold increase in viability and ~1.80-fold decrease in apoptosis relative to cells treated with only 5F12 (Figure 4A,B). Similarly, H1299 cell viability increased 2.00-fold and apoptosis decreased 1.95-fold in cells treated with 5F12 and IGF-1 compared to 5F12 treatment alone. 

Unlike the observed diminished effects of P-IGFBP-3 on IGF-1 signaling compared to IGFBP-3, no significant difference was observed on either cell viability or apoptosis in A549 and H1299 cells co-treated with 5F12, IGF-1, and either IGFBP-3 or P-IGFBP-3 in the absence or presence of cisplatin (Figure 4). These results might suggest that the effects observed in the absence of 5F12 reflect not only binding of IGFBP-3/P-IGFBP-3 to IGF-1 but also better inhibition of HA-CD44 signaling by IGFBP-3 than P-IGFBP-3, an interpretation supported by our findings showing no difference in the affinity of either IGFBP-3 or P-IGFBP-3 to IGF-1 (Figure 2C) but reduced binding of P-IGFBP-3 to HA (Figure 2B) in vitro. 

### 3.5. Sensitivity of A549 and H1299 Cells to Cisplatin Increased upon Co-Treatment with TBB and 5F12

**Figure 5:**
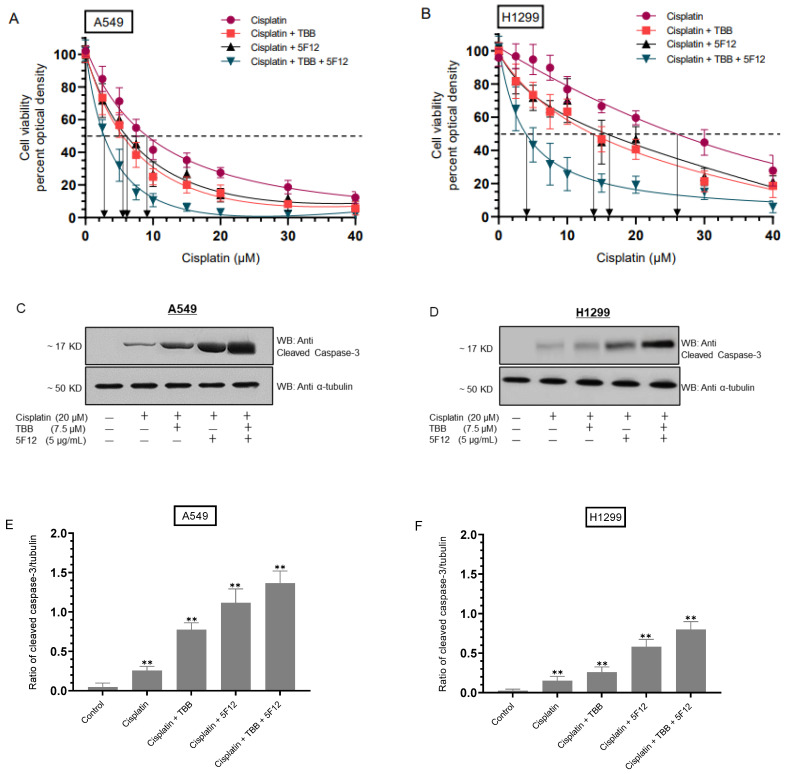
Co-treatment with TBB and 5F12 increased cell sensitivity to cisplatin**.** Cells (0.2 × 10^5^) were grown in 10% FBS-supplemented media for 24 h then serum starved overnight. The cell monolayers were next incubated in serum-free media for 72 h in the absence or presence of TBB (7.5 µM), 5F12 (5 μg/mL) added 2 h prior to addition of other treatments, cisplatin, and in combination then the cell viability (**A,B**) was measured as described in the Methods section. Optical densities (570 nm) were normalized for the curves by expressing each point relative to the best fitted Emax value (set to 100%). The data were then plotted as a function of increasing cisplatin concentrations and fit using the GraphPad Prism 9.4.1 software with a nonlinear regression curve fitting approach. Data were expressed as the mean ± S.D. of three independent experiments, each carried out in triplicate. Western blot analysis using anti-cleaved caspase-3 antibodies was also carried out (**C**,**D**) along with quantitation of cleaved caspase-3 levels (**E**,**F**) using Image J 1.47 v software. Data (**E**,**F**) are presented as mean ± SD, *n* = 3, ** *p* < 0.005 vs. the control group, Mann–Whitney test.

Our results show that treatment of A549 and H1299 cells with 5F12 increased sensitivity to cisplatin (Figure 4). Therefore, we examined the effect of 5F12 on A549 and H1299 cell viability with or without TBB as a function of increasing concentrations of cisplatin (Figure 5A,B). Cells were grown in 10% FBS-supplemented media for 24 h then serum starved overnight. The cell monolayers were next incubated in serum-free media for 72 h in the absence or presence of TBB, 5F12, or in combination as a function of increasing cisplatin concentrations. Cell viability (Figure 5A,B) was then measured as described in the Methods section. 

Treatment with TBB enhanced A549 cell sensitivity to cisplatin (IC_50_ values were 5.5 ± 0.95 μM vs. 9.1 ± 1.6 μM with cisplatin alone) and H1299 cell sensitivity to cisplatin (IC_50_ values were 13.9 ± 2.3 μM vs. 26.0 ± 4.5 μM with cisplatin alone). Addition of 5F12 also increased A549 cell sensitivity to cisplatin with an IC_50_ of 6.1 ± 0.98 μM, a value that was further decreased to 2.5 ± 0.42 μM in cells treated with TBB and 5F12 (Figure 5A). The trends were similar for H1299 cells with an IC_50_ value of 16.1 ± 2.6 μM for cells treated with 5F12 and 4.0 ± 0.64 μM for cells treated with 5F12 and TBB (Figure 5B). 

We also assessed the apoptotic effects of cisplatin in the absence or presence of TBB and 5F12 on A549 and H1299 cells, through examination of the activation of caspase-3 using Western blotting (Figure 5C,D). While the trend of caspase-3 activation was comparable in both cell lines, the ratio of cleaved caspase-3/tubulin (Figure 5E,F) was found to be higher in A549 cells than in H1299 cells. This ratio in A549 cells was ~0.25 when using cisplatin, ~0.85 with cisplatin/TBB, ~1.15 with cisplatin/5F12, and ~1.60 with cisplatin/TBB/5F12 while this ratio in H1299 cells was ~0.15 when using cisplatin, ~0.25 with cisplatin/TBB, ~0.55 with cisplatin/5F12, and ~0.80 with cisplatin/TBB/5F12.

### 3.6. A549 and H1299 Cell Co-Treatment with 5F12 and TBB Was Most Effective at Abolishing the PI3K and AKT Activities and the Phospho/Total NFκB Ratio and at Increasing the p53 Activity in A549 Cells Only

HA activation of CD44 is known to exert significant effects on cancer metastasis of a variety of tumor types including lung adenocarcinoma, in part, by potentiating the activation of PI3K, AKT, and nuclear localization of NFκB [18,19,21,23,74,75].

PI3K catalyzes phosphorylation of phosphatidylinositol 4,5-bisphosphate (PIP2) to generate the lipid second messenger phosphatidylinositol-3,4,5-triphosphate (PIP3) leading to recruitment and activation of multiple downstream signaling proteins that include the serine/threonine protein kinase, AKT [76,77]. The PI3K/AKT signaling network, known to be aberrantly activated in a number of human cancers, has diverse downstream effects driving multiple cellular processes critical for tumorigenesis including growth, survival, and proliferation [76,78]. 

Earlier reports have provided a link and crosstalk between AKT and NFκB [77,79,80]. NFκB activity was found to be essential for the PI3K and AKT induced oncogenic transformation while AKT induced-phosphorylation led to NFκB translocation into the nucleus, thereby regulating the transcriptional activity and tumorigenic functions of NFκB [77,79,80]. 

HA-CD44 signaling is known to increase AKT activation, and altering the activity and expression of p53 terminating its genomic surveillance response [81]. Upregulation of p53 was shown upon exposure of hepatocarcinoma cells to 4-MU [82]. and treatment of human chronic myelogenous leukemia cells, K562, with 4-MU was shown to regulate apoptosis by enhancing p53 expression [31]. Using A549 and H1299 cells, we previously found that IGFBP-3 blocks HA-CD44 signaling via a mechanism that depends on both p53 and acetylcholinesterase [57]. We also showed that blocking HA–CD44 signaling decreased the levels of heparanase in the media of both A549 and H1299 cell lines and increased p53 activity and the levels of IGFBP-3 in A549 cell media [67].

The majority of clinical studies suggest that mutations in the p53 tumor suppressor gene, common in lung adenocarcinoma [83]. and occur in ~34% of NSCLC patients, carry a poor prognosis and greater resistance to chemotherapy [84,85,86,87]. Recently, p53, known to function as a negative regulator of PI3K gene transcription, was shown to regulate the fate of NSCLC cells by blocking EGFR/PI3K/AKT signaling through crosstalk with AKT via feedback loops [84]. AKT was also reported to downregulate p53 conferring NSCLC resistance [84].

NFκB and p53 have an antagonistic relationship and opposing effects in cancer cells [88]. Through cross-regulation, both p53 and NFκB suppress each other’s ability to increase gene expression [88]. Increased NFκB activity has been found in p53-null mice and p53 loss was reported to promote activation of NFκB in a mouse model of KrasG12D-driven lung adenocarcinoma, while restoring p53 in p53-null lung tumors blocked NFκB activation and led to tumor suppression [89,90].

The ability of CK2 to promote tumorigenesis stems, in large part, from its ability to modulate key molecules and pathways responsible for proliferation and survival in different cell types, such as the NFκB and the PI3K/AKT signaling cascade [7,16,54]. Dysregulation of the p53 pathway has been reported to involve CK2 which can directly phosphorylate p53 and block its DNA binding activity or indirectly affect p53 activity through phosphorylation of p53 regulators [7,54]. 

To examine the effect of blocking CK2 and/or CD44 signaling on PI3K/AKT, NFκB, and p53, A549 and H1299 cells were grown in 10% FBS-supplemented media for 24 h, serum-starved overnight, then treated as indicated for 72 h with the inhibitors, TBB and/or 5F12. The PI3K activity was assayed by the Total In-Cell ELISA Kit and the AKT, NFκB, and p53 activities were measured on the same amount of protein of the cell lysate as described in the Methods section. 

**Figure 6:**
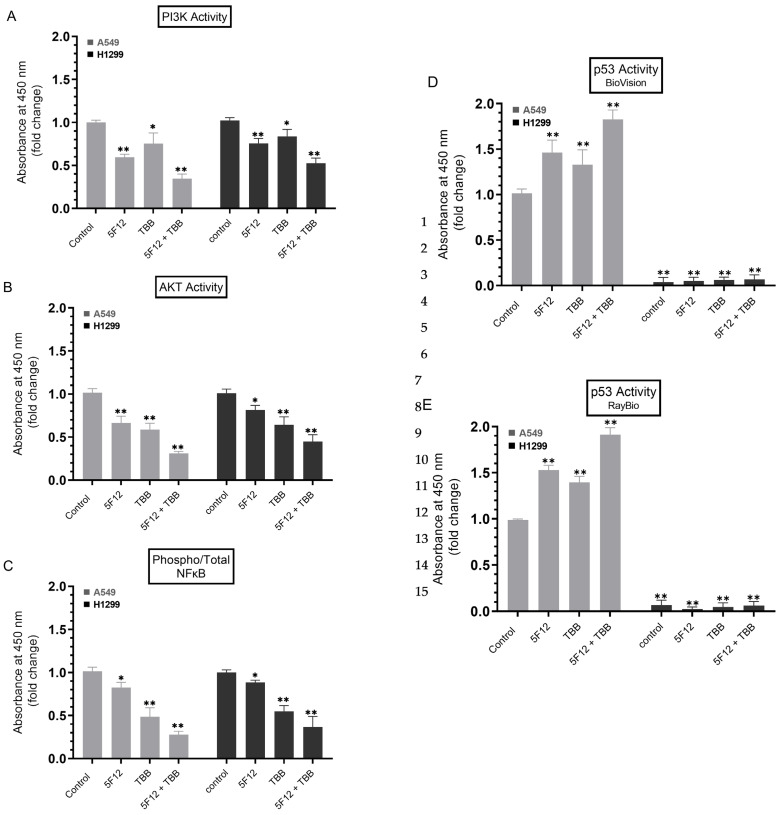
Co-treatment with 5F12 and TBB was most effective at decreasing the PI3K and AKT activities and the phospho/total NFκB ratio in both cell lines and at increasing the p53 activity in A549 cells only. Cells (0.2 × 10^5^) were grown in 10% FBS-supplemented media for 24 h. The following day, the cell monolayers were incubated in serum-free media for 24 h, then treated as indicated for 72 h with the inhibitors, TBB (7.5 µM) and/or 5F12 (5 μg/mL). The PI3K activity was assayed by the Total In-Cell ELISA Kit and the AKT, NFκB, and p53 activities were measured on the same amount of protein (3 µL of 600 µg/mL total protein) of the cell lysate as described in the Methods section. Data from five independent assays, each carried out in triplicate, were averaged, normalized, and expressed as fold change relative to cells not treated with inhibitors (Control, **A**–**C**) or to A549 control (**D**,**E**). The graphs summarize the results expressed as means ± SD (*n* = 5). Asterisks indicate a statistically significant difference from the control using the GraphPad 9.4.1 software, Mann–Whitney test. Statistical differences between different groups were analyzed by an ordinary one-way analysis of variance (ANOVA) followed by Tukey’s post hoc multiple comparison test, * *p* < 0.05, ** *p* < 0.0l.

A549 cell treatment with 5F12 or TBB resulted in ~1.65-fold decrease and ~1.35-fold decrease in PI3K activity, respectively, while co-treatment with both 5F12 and TBB led to a more pronounced decrease (~2.85-fold) in the activity of the kinase (Figure 6A). A similar trend but more modest effects were found using H1299 cells. Co-treatment of H1299 cells with TBB and 5F12 resulted in ~1.85-fold decrease in PI3K activity compared to treatment with either 5F12 (~1.30-fold decrease) or TBB (~1.20-fold decrease) (Figure 6A). Comparable decreases in the activity of AKT and NFκB were also found in both cell lines (Figure 6B,C). Treatment of A549 cells with 5F12 and TBB increased the activity of p53 ~1.45-fold and ~1.35-fold, respectively (Figure 6D). Upon co-treatment with both 5F12 and TBB, p53 activity was increased ~1.85-fold (Figure 6D). No effects on the activity of p53 were observed by treating H1299 cells with any of the inhibitors which is not surprising since these cells are p53-null [85].

### 3.7. Cisplatin Sensitivity Increased upon Co-Treatment of Cells with Inhibitors Targeted against PI3K, AKT, or NFκB but Decreased in A549 Cells Treated with the p53 Inhibitor 

Our results (Figure 1) show that cell viability decreased to a greater extent when cells were co-treated with TBB and cisplatin as opposed to treatment with either TBB or cisplatin. Similarly, treatment with a combination of 5F12 and cisplatin, as compared to the individual treatments, was more effective at decreasing cell viability (Figure 3A,C) and increasing apoptosis (Figure 3B,D). Our data also show increased A549 and H1299 cell sensitivity to cisplatin upon co-treatment with cisplatin, TBB, and 5F12 (Figure 5). Furthermore, blocking CK2 activity using TBB or CD44 signaling with the antibody, 5F12, alone or in combination decreased the activities of PI3K, AKT, and NFκB in both cell lines and increased p53 activation in A549 cells only (Figure 6). 

**Figure 7:**
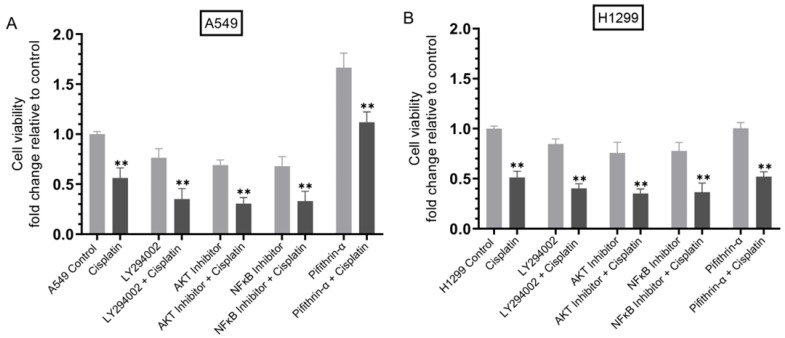
Cell sensitivity to cisplatin increased upon co-treatment of cells with inhibitors against PI3K, AKT, and NFκB but decreased in A549 cells treated with the p53 inhibitor. Cells (0.2 × 10^5^) were grown in 10% FBS-supplemented media for 24 h. The following day, the cell monolayers were incubated in serum-free media for 24 h, then treated as indicated for 72 h with inhibitors targeted against PI3K (LY294002, 14.5 μM), AKT (AKT inhibitor, 1.75 μM), NFκB (NFκB inhibitor, 18 μM), p53 (pifithrin-α, 10 μM), without or with cisplatin (10 μM when using A549 cells and 30 μM when using H1299 cells). Viability of A549 (A) and H1299 (B) cells was then determined as described in the Methods section. Data from five independent assays, each carried out in triplicate, were averaged, normalized, and expressed as fold change relative to untreated cells (Control) using the GraphPad 9.4.1 software. The graphs summarize the results expressed as means ± SD (*n* = 5). Asterisks indicate a statistically significant difference from the corresponding samples without cisplatin treatment for each cell line, Mann–Whitney test, ** *p* < 0.0l.

To examine cell sensitivity to cisplatin upon co-treatment with inhibitors, cells were grown in 10% FBS-supplemented media for 24 h then serum starved overnight. The cells were then treated as indicated for 72 h with inhibitors targeted against PI3K, AKT, NFκB, and p53, with or without cisplatin (Figure 7). Cell viability was then determined as described in the Methods section. 

Treatment of cells with cisplatin decreased cell viability of A549 and H1299 cells ~2.00-fold (Figure 7). Incubation of cells with the PI3K inhibitor, LY294002, decreased A549 cell viability ~1.30-fold in the absence of cisplatin and ~2.85-fold in the presence of cisplatin while H1299 cell viability was diminished ~1.15-fold and ~2.50-fold in the absence or presence of cisplatin, respectively. Similar trends were observed for both cell lines treated with inhibitors targeted against AKT and NFκB in the absence or presence of cisplatin (Figure 7). While no effects were observed on cell viability upon treatment of the p53-null cell line, H1299, with the p53 inhibitor, pifithrin-α (Figure 7B), A549 cells treated with this inhibitor led to ~1.65-fold increase in cell viability that was decreased to ~1.15-fold with cisplatin co-treatment (Figure 7A).

## 4. Discussion

Globally, lung cancer is the leading cause of cancer-related deaths and one of the most devastating malignancies, with NSCLC accounting for ~85% of all lung cancer mortalities [1]. Despite several therapeutic advances over the years, platinum-based chemotherapy currently remains the first-line chemotherapeutic option in the treatment of NSCLC with cisplatin being the most widely used platinum agent [2,3]. Patients’ clinical response is often compromised, however, by drug resistance leading to tumor relapse and chemotherapeutic failure [2]. Therefore, much remains unknown regarding the basic operative mechanisms underlying cisplatin resistance, a major clinical obstacle, which underscores the need for more research into these mechanisms.

In normal cells, the expression levels of CK2 are known to be well-regulated while overexpression of the kinase is frequently found in a number of human cancers, including lung, that can lead to tumor development [15]. CK2 intervenes in signaling cascades reported to play a role in evading drug response such as PI3K/AKT, NFκB, and p53 [7,16,52,54]. Upon inhibition, CK2 has been shown to display synergistic effects in reducing tumor growth when used in combination with drugs such as cisplatin [7,52]. Blocking CK2 activity was shown previously to reverse cisplatin resistance in cisplatin-resistant A549 cells [53]. Our results show that treatment with the CK2 inhibitor, TBB, (Figure 1A) or cisplatin (Figure 1B) decreased viability in a dose-dependent manner, both in A549 and H1299 cell lines. Treatment with TBB and cisplatin (Figure 1C,D) decreased cell viability more effectively than treatment with either TBB or cisplatin. 

IGFBP-3 expression was found earlier to be significantly diminished in cisplatin-resistant lung cancer cells [49,51,91]. Silencing of IGFBP-3 expression by promoter hypermethylation in cisplatin-treated tumor cells was reported previously to activate the PI3K/AKT pathway via de-repression of IGF-1R signaling, decreasing tumor cell sensitivity to cisplatin in NSCLC and inducing cisplatin-resistance in NSCLC patients [91]. IGFBP-3 knockout was shown to promote lung tumorigenesis while IGFBP-3 overexpression led to increased apoptosis, decreased cell growth, and increased susceptibility of NSCLC cell lines to cisplatin treatment [49,91]. 

IGFBP-3 is known to be phosphorylated by CK2 [12,13]. Our results show that phosphorylation of IGFBP-3 by CK2 decreases its binding to HA (Figure 2A,B). CK2 phosphorylation of IGFBP-3 was found to block interactions of IGFBP-3 with the acid-labile subunit (ALS) [13,69]., known to bind residues 228–232 of IGFBP-3 which comprise part of the 18-residue basic domain (^215^-KKGFYKKKQCRPSKGRKR-^232^), previously reported to interact with either humanin [70]. or HA [50,71]. CK2 phosphorylation of IGFBP-3 was thought to modulate interactions of IGFBP-3 with ALS and the cell surface, both of which mediated by the basic carboxyl-terminal residues of IGFBP-3 [12,13]. Blocking binding of phosphorylated IGFBP-3 by CK2 to HA may be a consequence of an increase in the net negative charge of IGFBP-3 due to phosphorylation that may diminish its interaction with HA. No effects of CK2 phosphorylation of IGFBP-3 were observed on its binding to IGF-1 (Figure 2C). The lack of the observed difference in the binding of either IGFBP-3 or P-IGFBP-3 to IGF-1, is consistent with previously published reports showing that CK2 phosphorylation of IGFBP-3 is not essential for the ability of IGFBP-3 to bind IGF-1 [13,69]. 

Binding of HA to its main receptor, CD44, has been reported to promote cell survival pathways [75,92,93,94,95]. CD44 overexpression has been linked to drug resistance in lung cancer with CD44+ lung cancer cells being more resistant to cisplatin than CD44− cells [96,97,98]. Therefore, unravelling mechanisms to decrease CD44 signaling might be a beneficial approach in targeting cisplatin resistance. Previously, we showed that binding of IGFBP-3 to HA through residues 215–232 in the C-terminal region of the protein blocks interactions of HA with its receptor, CD44, decreasing A549 cell viability [50]. We later found that blocking HA-CD44 signaling with IGFBP-3 involves a mechanism that depends on both p53 and acetylcholinesterase and is IGF-independent [57]., and that interaction of IGFBP-3 with HA was not modulated by glycosylation or reduction of IGFBP-3 [55]. More recently, we found decreased levels of heparanase in the media of both A549 and H1299 cell lines and increased p53 activity and the levels of IGFBP-3 in A549 cell media upon blocking HA–CD44 signaling [67]. In this study, our results show that phosphorylation of IGFBP-3 by CK2 rendered IGFBP-3 less effective than the non-phosphorylated protein at reducing cell viability or increasing apoptosis in the absence or presence of cisplatin (Figure 3). These results are consistent with previous observations showing that blocking activation of CK2 enhances IGFBP-3 induced apoptosis [12]., while apoptosis induced by IGFBP-3 was partially blocked by CK2-mediated phosphorylation. We also reported, previously, that IGFBP-3 likely exerts its cytotoxic effects on cell survival through a mechanism that depends on HA-CD44 molecular interactions since cell co-treatment with IGFBP-3 and the anti-CD44 antibody (5F12), known to be antagonistic towards HA-CD44 binding, did not have an additive negative effect on cell viability [50]. In this study, our results (Figure 3) suggest that blocking CD44 signaling by 5F12 augments the effects of cisplatin and that IGFBP-3 likely operates via a mechanism involving CD44.

In examining the effects of IGF-1 on cell viability or apoptosis in the absence or presence of IGFBP-3 or CK2-phosphorylated IGFBP-3, we found that co-treatment of A549 and H1299 cells with IGF-1 and IGFBP-3 was more effective at decreasing cell viability and increasing apoptosis than co-treatment of cells with IGF-1 and P-IGFBP-3 with or without cisplatin (Figure 4). Our results (Figure 2C) show that phosphorylation of IGFBP-3 by CK2 does not affect its binding to IGF-1, therefore, the effect on cell viability or apoptosis (Figure 4) must involve other mechanisms at play besides binding of either IGFBP-3 or P-IGFBP-3 to IGF-1. No difference in cell viability or apoptosis was found upon cell treatment with IGF-1 and either IGFBP-3 or P-IGFBP-3 in the presence of the CD44 antibody, 5F12, with or without cisplatin (Figure 4). Our findings showed no difference in the affinity of either IGFBP-3 or P-IGFBP-3 to IGF-1 (Figure 2C) but reduced binding of P-IGFBP-3 to HA (Figure 2B) in vitro. A likely explanation for the results obtained in the absence of 5F12 (Figure 4) might be that both IGFBP-3 and P-IGFBP-3 can bind IGF-1 and neutralize its effect on cell viability or apoptosis, but that IGFBP-3 is more efficient than P-IGFBP-3 at binding HA and blocking HA-CD44 signaling.

Co-treatment of A549 and H1299 cells with TBB and 5F12 increased cisplatin sensitivity (Figure 5) and more effectively blocked the PI3K and AKT activities and the phospho/total NFκB ratio (Figure 6) pointing to the effectiveness of combining different treatments to increase cell sensitivity to cisplatin. Conversely, the p53 activity increased in A549 cells treated with TBB and 5F12 while no effects were observed on the activity of p53 by treating H1299 cells with any of the inhibitors which is not surprising since H1299 cells are p53-null (Figure 6) [85].

**Figure 8:**
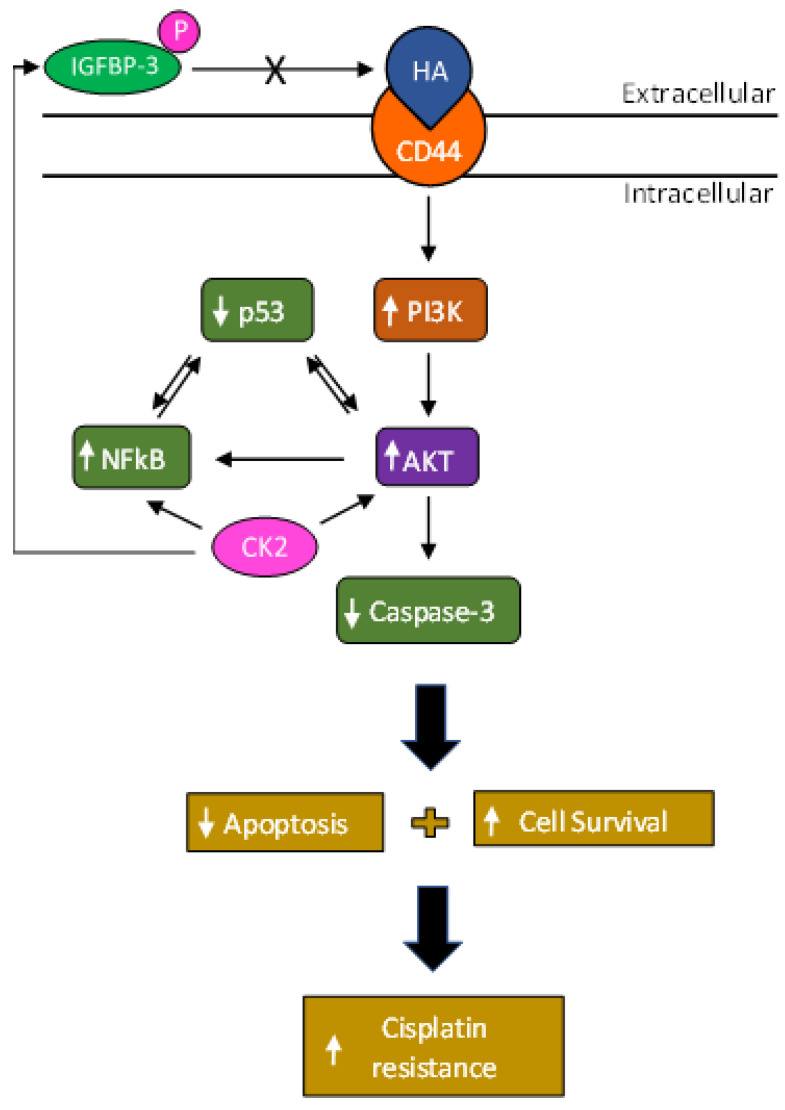
Representation of the main hypothesis and findings of this study. Phosphorylation of IGFBP-3 by CK2 blocks its interaction with HA, enabling HA-CD44 signaling which in turn leads to activation of PI3K/AKT/NFκB signaling and inhibition of p53. This results in inhibition of apoptosis and increased cell survival and chemoresistance. In this model, CK2 further increases chemoresistance by activating AKT and NFκB.

PI3K catalyzes the conversion of PIP2 to PIP3 which then leads to AKT activation that in turn downregulates apoptosis, increasing cell proliferation, tumor survival, and metastasis [76]. A key role for the PI3K/AKT pathway has been demonstrated in the development of cancer cell resistance to cisplatin among other chemotherapeutic agents [27,99]. Tumorigenicity was reported to decrease upon blocking the activity of NFκB [80]. Suppression of NFκB activity in A549 cells was shown to sensitize the cells to cisplatin-induced cytotoxicity [100]. Previous published findings provided evidence for the role of the PI3K–NFκB axis in NSCLC cisplatin resistance and suggested that inhibitors targeted against NFκB could provide a promising treatment strategy for patients with NSCLC [101]. Cisplatin resistance can be caused by loss of apoptosis regulation [102]. Several reports suggest that loss of normal p53 function can lead to NSCLC oncogenesis and tumor progression [85,90,103,104,105,106]. Mutations of the p53 gene are highly prevalent and frequent molecular events in cancer, found in ~60% of NSCLC cases with correlation to poor prognosis [85,106,107]. The tumor suppressor gene, p53, is commonly mutated in human cancers and upon DNA damage by, e.g., cisplatin, has been shown to activate apoptosis [5,84]. Aberrant expression of p53 and cancer cells with mutated p53 showed reduced susceptibility to apoptosis and linked to cisplatin-resistance in NSCLC [5]. Despite the basal level of p53 expression, increasing the concentration of p53 correlated positively with increased effects of cisplatin in NSCLC and cytotoxic response [108]. Consistent with these reports, we found increased cell sensitivity to cisplatin upon co-treatment with inhibitors targeted against PI3K, AKT, and NFκB but decreased cisplatin sensitivity in A549 cells treated with the p53 inhibitor (Figure 7). Based on our findings, we propose a model (Figure 8) depicting regulation of cisplatin resistance in lung cancer cells by CK2, CD44, and IGFBP-3. While no information can be provided from our data in this study as to the intracellular and/or extracellular phosphorylation of IGFBP-3 by CK2 in A549 cells, research is currently ongoing in our laboratory to address this objective. 

Unravelling molecular mechanisms that cause cellular resistance to anticancer drugs is crucial for the development of novel strategies for the therapeutic treatment of lung cancer. Our results from this study support the possible relevance of blocking phosphorylation of IGFBP-3 by CK2 to reverse cisplatin resistance in NSCLC cells and might lead to effective and novel therapeutic approaches to enhance the efficacy of cisplatin in NSCLC.

## Data Availability

Not applicable.
